# Auswirkungen der SARS-CoV‑2-Pandemie auf die universitäre Hals-Nasen-Ohren-Heilkunde im Bereich der Forschung, Lehre und Weiterbildung

**DOI:** 10.1007/s00106-021-01001-8

**Published:** 2021-01-27

**Authors:** T. Stöver, S. Dazert, S. K. Plontke, S. Kramer, P. Ambrosch, C. Arens, C. Betz, D. Beutner, C. Bohr, K.-L. Bruchhage, M. Canis, A. Dietz, O. Guntinas-Lichius, R. Hagen, W. Hosemann, H. Iro, J. P Klussmann, A. Knopf, S. Lang, M. Leinung, T. Lenarz, H. Löwenheim, C. Matthias, R. Mlynski, H. Olze, J. Park, P. Plinkert, A. Radeloff, N. Rotter, C. Rudack, A. Bozzato, J. Schipper, M. Schrader, P. J. Schuler, S. Strieth, B. A. Stuck, S. Volkenstein, M. Westhofen, G. Wolf, B. Wollenberg, T. Zahnert, J. Zenk, T. K. Hoffmann

**Affiliations:** 1 Klinik für Hals-Nasen-Ohrenheilkunde, Kopf- und Halschirurgie, Universitätsklinikum Frankfurt a.M., Frankfurt a.M., Deutschland; 2 Klinik für Hals-Nasen-Ohrenheilkunde, Ruhr-Universität-Bochum, St. Elisabeth-Hospital, Bochum, Deutschland; 3 Klinik für Hals-Nasen-Ohrenheilkunde, Kopf- und Halschirurgie, Universitätsklinikum Halle/S., Halle/S., Deutschland; 4 Klinik für Hals-Nasen-Ohrenheilkunde, Kopf- und Halschirurgie, Universitätsklinikum Kiel, Kiel, Deutschland; 5 Klinik für Hals-Nasen-Ohrenheilkunde, Kopf- und Halschirurgie, Universitätsklinikum Magdeburg, Magdeburg, Deutschland; 6 Klinik und Poliklinik für Hals‑, Nasen- und Ohrenheilkunde, Universitätsklinikum Hamburg Eppendorf, Hamburg, Deutschland; 7 Klinik für Hals-Nasen-Ohrenheilkunde, Universitätsklinikum Göttingen, Göttingen, Deutschland; 8 Klinik für Hals-Nasen-Ohrenheilkunde, Universitätsklinikum Regensburg, Regensburg, Deutschland; 9 Klinik für Hals-Nasen-Ohrenheilkunde, Universitätsklinikum Lübeck, Lübeck, Deutschland; 10 Klinik und Poliklinik für Hals-Nasen-Ohrenheilkunde, Klinikum der Universität München, LMU München, München, Deutschland; 11 Klinik für Hals-Nasen-Ohrenheilkunde, Universitätsklinikum Leipzig, Leipzig, Deutschland; 12 Klinik für Hals-Nasen-Ohrenheilkunde, Universitätsklinikum Jena, Jena, Deutschland; 13 Klinik für Hals-Nasen-Ohrenheilkunde, Universitätsklinikum Würzburg, Würzburg, Deutschland; 14 Klinik für Hals-Nasen-Ohrenheilkunde, Kopf- und Halschirurgie, Universitätsklinikum Greifswald, Greifswald, Deutschland; 15 Klinik für Hals-Nasen-Ohrenheilkunde, Kopf- und Halschirurgie, Universitätsklinikum Erlangen, Erlangen, Deutschland; 16 Klinik für Hals-Nasen-Ohrenheilkunde, Uniklinik Köln und Medizinische Fakultät, Universität zu Köln, Köln, Deutschland; 17 Klinik für Hals-Nasen-Ohrenheilkunde, Universitätsklinikum Freiburg, Freiburg, Deutschland; 18 Klinik für Hals-Nasen-Ohrenheilkunde, Universitätsklinikum Essen, Essen, Deutschland; 19 Klinik für Hals-Nasen-Ohrenheilkunde, Medizinische Hochschule Hannover, Hannover, Deutschland; 20 Klinik für Hals-Nasen-Ohrenheilkunde, Kopf- und Halschirurgie, Universitätsklinikum Tübingen, Tübingen, Deutschland; 21 Klinik für Hals-Nasen-Ohrenheilkunde, Universitätsklinikum Mainz, Mainz, Deutschland; 22 Klinik und Poliklinik für Hals-Nasen-Ohrenheilkunde, Kopf- und Halschirurgie „Otto Körner“, Universitätsmedizin Rostock, Rostock, Deutschland; 23 Klinik für Hals-Nasen-Ohrenheilkunde, Charité – Universitätsmedizin Berlin, Berlin, Deutschland; 24 Klinik für Hals-Nasen-Ohrenheilkunde, Universität Witten/Herdecke, Witten/Herdecke, Deutschland; 25 Klinik für Hals-Nasen-Ohrenheilkunde, Universitätsklinikum Heidelberg, Heidelberg, Deutschland; 26 Klinik für Hals-Nasen-Ohrenheilkunde, Universitätsklinikum Oldenburg, Oldenburg, Deutschland; 27 Klinik für Hals-Nasen-Ohrenheilkunde, Kopf- und Halschirurgie, Universitätsklinikum Mannheim, Mannheim, Deutschland; 28 Klinik für Hals-Nasen-Ohrenheilkunde, Universitätsklinikum Münster, Münster, Deutschland; 29 Klinik für Hals-Nasen-Ohrenheilkunde, Universitätsklinikum des Saarlandes, Saarlandes, Deutschland; 30 Klinik für Hals-Nasen-Ohrenheilkunde, Universitätsklinikum Düsseldorf, Düsseldorf, Deutschland; 31 Klinik für Hals-Nasen-Ohrenheilkunde, Kopf- und Halschirurgie, Universitätsklinikum Minden, Minden, Deutschland; 32 Klinik für Hals-Nasen-Ohrenheilkunde, Kopf- und Halschirurgie, Universitätsklinikum Ulm, Ulm, Deutschland; 33 Klinik und Poliklinik für Hals-Nasen-Ohren-Heilkunde, Universitätsklinikum Bonn, Bonn, Deutschland; 34 Klinik für Hals‑, Nasen- und Ohrenheilkunde, Universitätsklinikum Gießen und Marburg GmbH, Standort Marburg, Philipps-Universität Marburg, Marburg, Deutschland; 35 Klinik für Hals-Nasen-Ohrenheilkunde, Kopf- und Halschirurgie, Universitätsklinikum Aachen, Aachen, Deutschland; 36 Klinik für Hals-Nasen-Ohrenheilkunde, Universitätsklinikum Gießen und Marburg GmbH, Standort Gießen, Justus-Liebig-Universität, Gießen, Deutschland; 37 Klinik für Hals-Nasen-Ohrenheilkunde, Klinikum rechts der Isar der Technischen Universität München, München, Deutschland; 38 Klinik für Hals-Nasen-Ohrenheilkunde, Universitätsklinikum Dresden, Dresden, Deutschland; 39 Klinik für Hals-Nasen-Ohrenheilkunde, Universitätsklinikum Augsburg, Augsburg, Deutschland; 40 Helios Hanseklinikum Stralsund, Stralsund, Deutschland; 41 Evangelisches Krankenhaus Düsseldorf, Düsseldorf, Deutschland

**Keywords:** COVID-19, SARS-CoV‑2-Pandemie, Universitätskliniken, Hals-Nasen-Ohren-Heilkunde, HNO, Forschung, Lehre, Weiterbildung, COVID-19, SARS-CoV‑2 pandemic, University hospitals, Otorhinolaryngology, ORL, Research, Teaching, Specialist training, Residency

## Abstract

**Hintergrund:**

Ab Frühjahr 2020 kam es zur weltweiten Verbreitung von SARS-CoV‑2 mit der heute als erste Welle der Pandemie bezeichneten Phase ab März 2020. Diese resultierte an vielen Kliniken in Umstrukturierungen und Ressourcenverschiebungen. Ziel unserer Arbeit war die Erfassung der Auswirkungen der Pandemie auf die universitäre Hals-Nasen-Ohren(HNO)-Heilkunde für die Forschung, Lehre und Weiterbildung.

**Material und Methoden:**

Die Direktorinnen und Direktoren der 39 Universitäts-HNO-Kliniken in Deutschland wurden mithilfe einer strukturierten Online-Befragung zu den Auswirkungen der Pandemie im Zeitraum von März bis April 2020 auf die Forschung, Lehre und die Weiterbildung befragt.

**Ergebnisse:**

Alle 39 Direktorinnen und Direktoren beteiligten sich an der Umfrage. Hiervon gaben 74,4 % (29/39) an, dass es zu einer Verschlechterung ihrer Forschungstätigkeit infolge der Pandemie gekommen sei. Von 61,5 % (24/39) wurde berichtet, dass pandemiebezogene Forschungsaspekte aufgegriffen wurden. Von allen Kliniken wurde eine Einschränkung der Präsenzlehre berichtet und 97,5 % (38/39) führten neue digitale Lehrformate ein. Im Beobachtungszeitraum sahen 74,4 % der Klinikdirektoren die Weiterbildung der Assistenten nicht gefährdet.

**Schlussfolgerung:**

Die Ergebnisse geben einen Einblick in die heterogenen Auswirkungen der Pandemie. Die kurzfristige Bearbeitung pandemiebezogener Forschungsthemen und die Einführung innovativer digitaler Konzepte für die studentische Lehre belegt eindrücklich das große innovative Potenzial und die schnelle Reaktionsfähigkeit der HNO-Universitätskliniken, um auch während der Pandemie ihre Aufgaben in der Forschung, Lehre und Weiterbildung bestmöglich zu erfüllen.

## Abstract (en)

**Background:**

From spring 2020, SARS-CoV‑2 began to spread worldwide, with what is now known as the first wave of the pandemic, starting in March 2020. This resulted in restructuring and shift of resources at many hospitals. The aim of our work was to detect the effects of the pandemic on the german Oto-Rhino-Laryngology (ORL) university hospitals in terms of research, student teaching and further specialist training.

**Material and methods:**

The chairmen of the 39 ORL university hospitals in Germany were asked about the effects of the pandemic on research, student teaching and ORL specialist training (residency) in the period from March to April 2020 using a structured online survey.

**Results:**

All 39 chairmen took part in the survey. Of these, 74.4% (29/39) stated that their research activities had deteriorated as a result of the pandemic. In 61.5% (24/39) pandemic-related research issues were addressed. All hospitals reported a restriction for in-house teaching and 97.5% (38/39) introduced new digital teaching methods. During the observation period, 74.4% of the chairmen did not see ORL specialist training (residency)at risk.

**Conclusion:**

Our results provide an insight into the heterogeneous effects of the pandemic. The fast processing of pandemic-related research topics and the introduction of innovative digital concepts for student teaching impressively demonstrates the great innovative potential and the ability of the ORL university hospitals to react quickly in order to maintain their tasks in research, student teaching and ORL specialist training in the best possible way even during the pandemic.

Die SARS-CoV-2-Pandemie [1] hat weltweit zu erheblichen Veränderungen des privaten und öffentlichen Lebens geführt und auch die medizinischen Fakultäten im Bereich der Krankenversorgung, Lehre und Forschung stark beeinflusst. Aktuell haben sich weltweit mehr als 80 Mio. Menschen mit dem Virus infiziert, mit ca. 1,8 Mio. tödlichen Verläufen (Stand Dezember 2020) [2]. Nach Beginn bzw. Erkennen der Pandemie in Deutschland im März 2020 wurden weitreichende Maßnahmen ergriffen, um die Ausbreitung des Virus einzudämmen. Die strengen Kontaktbeschränkungen hatten das öffentliche Leben zwischenzeitlich weitgehend zum Erliegen gebracht. Das gesamte Gesundheitssystem in Deutschland hatte sich auf ein großes Aufkommen von COVID-19-Patienten vorbereitet. In den Krankenhäusern beinhaltete diese Vorbereitung massive organisatorische und personelle Umstrukturierungen, die mit einer Umverteilung der vorhandenen Ressourcen einhergingen und auch die Universitätskliniken für Hals-Nasen-Ohren-Heilkunde, Kopf- und Halschirurgie betrafen (im weiteren Text werden diese Einrichtungen als „HNO-Universitätskliniken“ bezeichnet). Im Rahmen dieser weitreichenden Umstellungsmaßnahmen wurden auch die Vorgaben für Forschung und Lehraktivitäten an den Universitäten und damit auch speziell an den Universitätsklinika geändert. Der Beginn der Pandemie in Deutschland im März 2020 lag nur wenige Wochen vor dem Beginn des Sommersemesters. Die Mehrzahl der Präsenzveranstaltungen in der Lehre wurden abgesagt. Viele Forschungslabore und Studienzentralen blieben auf unbestimmte Zeit geschlossen, andere fokussierten ihre gesamte Kapazität auf die Erforschung des neuartigen Virus.

Um ein differenziertes Bild über die Auswirkungen der SARS-CoV-2-Pandemie auf die universitäre Hals-Nasen-Ohren-Heilkunde zu erhalten, wurde von April bis Mai 2020 eine strukturierte, internetbasierte Umfrage zu den Auswirkungen der Pandemie auf die Krankenversorgung, die Forschung und die Lehre unter den 39 HNO-Universitätskliniken durchgeführt. Die in Bezug zur Krankenversorgung stehenden Ergebnisse der Umfrage wurden bereits in einer separaten Arbeit publiziert [3]. Ziel der hier präsentierten Anteile der Studie war die Erfassung der Auswirkungen der SARS-CoV-2-Pandemie auf die Forschung, Lehre und Weiterbildung der Assistenzärzte in den ersten Wochen der Pandemie. Die Ergebnisse geben einen Einblick in die überwiegend negativen Auswirkungen der Pandemie, zeigen aber auch positive Effekte, z. B. im Hinblick auf die kurzfristige Bearbeitung pandemiebezogener Forschungsthemen oder die Einführung innovativer digitaler Konzepte für die studentische Lehre.

## Material und Methoden

Bei der vorliegenden Untersuchung handelt es sich um eine retrospektive, fragebogenbasierte Datenerhebung. Die Datenerfassung wurde mithilfe des Online-Befragungstools „SurveyMonkey“ (surveymonkey.com, SurveyMonkey, San Mateo, USA) durchgeführt. Sie bestand aus der Beantwortung von insgesamt 87 Fragen aus den Bereichen der allgemeinen Struktur der Einrichtung (7 Fragen), der Tätigkeit im Bereich Forschung, der studentischen Lehre (Ausbildung) und der Weiter- und Fortbildung (22 Fragen), der Krankenversorgung (39 Fragen) sowie der strukturellen Auswirkungen der SARS-CoV-2-Pandemie auf die jeweilige Einrichtung (19 Fragen). Die Antworten sollten sich ausschließlich auf den Beurteilungszeitraum von Mitte März bis Mitte April 2020 beziehen.

Die Antwortmöglichkeiten bestanden in Abhängigkeit von der jeweiligen Fragestellung in einer Ja/Nein-Option, einem Item vom Likert-Typ, einer Prozentangabe, einer absoluten Angabe eines Zahlenwerts oder der Auswahl aus einer vorgegebenen Liste verschiedener Antwortmöglichkeiten. Jede Frage enthielt auch die Option „keine Antwort“ („k. A.“). Verschiedene Fragen enthielten die Option, freie Anmerkungen abzugeben.

Der Online-Link zur Beantwortung der Fragen wurde per E‑Mail an die Leiterinnen und Leiter der 39 universitären Kliniken für Hals-Nasen-Ohren-Heilkunde in Deutschland versendet. Die Aussendung erfolgte am 27.04.2020. Die Befragung wurde nach 10 Tagen, am 06.05.2020, beendet. Die Beantwortung der Fragen erfolgte anonym, sodass eine Zuordnung der Daten zu Ursprungsorten der Einrichtung nicht möglich ist.

Die erhobenen Ergebnisse wurden entweder tabellarisch oder grafisch dargestellt. Sofern dies möglich war, wurden statistische Signifikanzen berechnet und dargestellt. Hierzu wurden der zweiseitige Wilcoxon-Rangsummentest (nichtparametrischer Test für verbundene Stichproben) und nach Prüfung auf Normalverteilung der „single-sample t‑test“ verwendet (SPSS®, Version 25, Fa. IBM Corp., Armonk, NY, USA).

Aufgrund der Menge der erfassten Daten und der verschiedenen Themenbereiche werden hier nur die Ergebnisse für die Fragen zu Forschung, studentischer Lehre und Weiterbildung berichtet. Die Fragen zur universitären HNO-ärztlichen Versorgung und zu klinischen Strukturen wurden separat ausgewertet und veröffentlicht [3].

Da es sich nicht um die Erhebung von patientenbezogenen Daten handelte, war kein Votum einer Ethik-Kommission erforderlich.

Die in dieser Arbeit verwendeten Formulierungen sind als gender-unspezifisch zu betrachten (z. B. „der Klinikdirektor“ ist geschlechtsneutral (m/w/d) zu verstehen). Zur besseren Lesbarkeit wird in der Regel eine einheitliche Form verwendet, die aber nicht die jeweilige Bezeichnung des Geschlechts der Person wiedergeben soll.

## Ergebnisse

Die Auswertung der Fragebögen ergab nachfolgend aufgeführte Ergebnisse zu den Bereichen Forschung, Lehre und Weiterbildung.

### Forschung

74,4 % (29/39) der befragten Klinikdirektoren gaben an, dass es für sie zu einer Verschlechterung ihrer Forschungstätigkeit infolge der Corona-Pandemie gekommen sei. 7,7 % (3/39) gaben keine Veränderung und 17,9 % (7/39) eine Verbesserung an.

**Figure 1:**
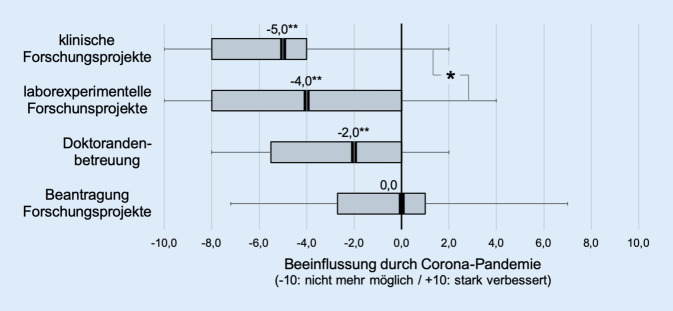

In 64,1 % (25/39) sei auch die Durchführung bereits laufender Forschungsprojekte gefährdet. Ergänzend wurden verschiedene Teilbereiche der Forschung auf einer Skala von −10 (gar nicht mehr möglich) bis +10 (stark verbessert) beurteilt. Die Ergebnisse zeigten, dass demnach klinische Forschungsprojekte (Median −5,0/Interquartilsabstand 4,0) von der Pandemie stärker negativ beeinflusst wurden als laborexperimentelle Projekte (−4,0/8,0). Dieser Unterschied war statistisch signifikant (*p* < 0,05). Die Betreuung von Doktoranden (−2/5,5) war etwas weniger stark beeinträchtigt. Auffällig für alle Teilbereiche war eine große Schwankungsbreite der Ergebnisse: Zwar lagen die Antworten im Mittel hochsignifikant unter dem Zustand vor dem Befragungszeitraum (*p* < 0,01), aber mehrfach wurden gleichwertige oder in Einzelfällen sogar verbesserte Forschungsbedingungen im Befragungszeitraum angegeben. Im Teilbereich „Beantragung von Forschungsprojekten“ zeigte sich dagegen keine signifikante Abweichung (Median 0,0 bei einem Interquartilsabstand von 3,5) (Abb. 1).

In 61,5 % (24/39) der Universitätskliniken haben sich die Forschungsthemen im Zeitraum der Beobachtung verändert. Im Fall einer Veränderung der Forschungsthemen hatten 91,7 % (22/24) zum Zeitpunkt der Erhebung bereits Forschungsvorhaben mit Bezug zur SARS-CoV-2-Pandemie begonnen. 66,7 % (26/39) der befragten Klinikdirektoren gaben an, dass sie planen, inhaltlich neue Forschungsanträge mit Bezug zur Corona-Thematik zu stellen.

18 von 39 Klinikdirektoren (46,2 %) gaben an, dass das Interesse und Engagement ihrer Weiterbildungsassistenten an einer Forschungstätigkeit zugenommen habe, und 19 von 39 (48,7 %) gaben an, dass dies gleichgeblieben sei. Bei 2 von 39 Teilnehmern der Umfrage (5,1 %) wurde dagegen über eine Abnahme des Forschungsinteresses der Assistenten berichtet.

Eine geänderte Nutzung von Forschungsflächen infolge der Pandemie wurde durch 3 von 39 Kliniken (7,7 %) angeben. 93,3 % (36/39) gaben keine geänderte Verwendung von Forschungsflächen an.

### Studentische Lehre

**Figure 2:**
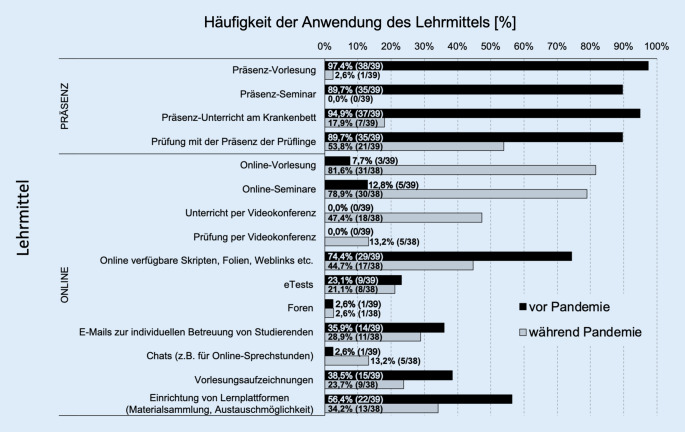

Durch alle 39 HNO-Universitätskliniken wurde eine Einschränkung der Präsenzlehre angegeben. 38 von 39 Klinikdirektoren (97,4 %) berichteten, dass infolge der SARS-CoV-2-Pandemie neue Lehrformate eingeführt wurden. Hierzu wurden Präsenzvorlesungen, Präsenzseminare und Unterricht am Krankenbett durch Online-Vorlesungen, -Seminare und -Unterricht ersetzt (Abb. 2).

Die Prüfungen wurden in 53,8 % (21/39) der Universitätskliniken auch während des Beobachtungszeitraums in Form einer Präsenzveranstaltung durchgeführt. Andere digitale Medien (online verfügbares Lehrmaterial, Vorlesungsaufzeichnungen) und Kommunikationswege (E-Mail, Lehrplattformen) wurden in einzelnen Kliniken bereits vor der Corona-Pandemie verwendet und zum Zeitpunkt der Befragung weiterbetrieben und ausgebaut (Abb. 2).

**Figure 3:**
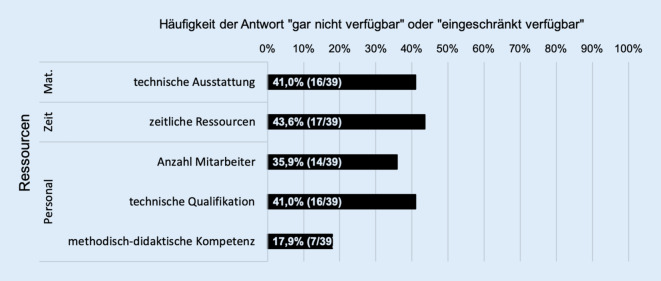

30,8 % (12/39) der befragten Klinikdirektoren gaben mangelnde Ressourcen zur Einführung neuer Lehrformate an. Die Einschränkungen betrafen etwa zu gleichen Teilen zeitliche (43,6 %; 17/39), technische (41,0 %; 16/39) und personelle Ressourcen (35,9 %; 14/39) sowie die technische Qualifikation der Mitarbeiter (41,0 %; 16/39). In 17,9 % (7/39) wurde eine fehlende methodisch-didaktische Kompetenz im Umgang mit den ungewohnten Lehrmedien benannt (Abb. 3).

### Ärztliche Weiterbildung

74,4 % (29/39) der Klinikdirektoren sahen zum Zeitpunkt der Befragung die erfolgreiche Weiterbildung ihrer Assistenzärzte für das Fach Hals-Nasen-Ohren-Heilkunde nicht gefährdet, während die verbleibenden 25,6 % (10/39) die Weiterbildung im Beurteilungszeitraum als nicht gewährleistet betrachteten.

**Figure 4:**
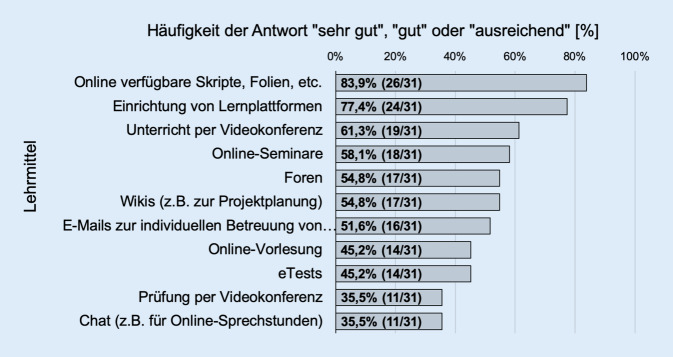

79,5 % (31/39) erachteten die Einführung neuer Lehr- und Unterrichtsformate für eine Anwendung im Bereich der Assistentenweiterbildung als grundsätzlich geeignet. Als nicht geeignet sahen dies 18 % (7/39) an (keine Angaben 2,6 %; 1/39). Als mögliche geeignete neue Lehr- und Unterrichtsformate für die Weiterbildung der Assistenten wurden insbesondere online verfügbares Lehrmaterial (83,9 %; 26/31), die Einrichtung von Lehrplattformen (77,4 %; 24/31), Unterricht per Videokonferenz (61,3 %; 19/31) und Online-Seminare genannt (58,1 %; 18/31) (Abb. 4).

## Diskussion

Die vorgestellte retrospektive Untersuchung hatte zum Ziel, anhand einer Online-Befragung der Klinikdirektoren aller universitären Kliniken für HNO-Heilkunde die Auswirkungen der SARS-CoV-2-Pandemie auf die 39 universitären HNO-Kliniken in Deutschland für die Aspekte Forschung, Lehre und Ausbildung der Weiterbildungsassistenten im Zeitraum vom 15. März bis 15. April 2020 zu untersuchen. In diesem Zeitraum lag die sog. erste Welle des exponentiellen Anstiegs von Neuinfektionen der SARS-CoV-2-Pandemie. Dieser Zeitraum war geprägt von der Erwartung einer drohenden Überlastung der Versorgungskapazitäten und einem befürchteten Massenanfall von COVID-19-Erkrankten. In einer früheren Arbeit konnten wir bereits die tiefgreifenden Auswirkungen der Pandemie auf die Krankenversorgung im genannten Beobachtungszeitraum darstellen [3]. Die hier präsentierten Ergebnisse demonstrieren die Auswirkungen im gleichen Zeitraum auf die Forschung, die Lehre, aber auch auf die Weiterbildung der Assistenzärzte, die zu den besonderen Aufgaben der 39 universitären HNO-Kliniken in Deutschland zählt.

Zunächst sollen die Auswirkungen auf den Bereich der Forschung betrachtet werden. Hierbei ließ sich eine heterogene Beurteilung erheben. Während fast 75 % der Kliniken eine Verschlechterung der Voraussetzungen für ihre Forschungstätigkeit beschrieben, gaben ca. 25 % keine Veränderung oder sogar eine Verbesserung der Forschungsaktivitäten an. Dieses Ergebnis erscheint zunächst überraschend, muss aber vor dem Hintergrund der Situation im März und April 2020 betrachtet werden. In diese Phase der Pandemie fiel eine drastische Reduktion der elektiven Operationen und der klinischen Untersuchungskapazitäten im Nachgang zu dem an die Kliniken gerichteten Rundschreiben von Bundesgesundheitsminister Jens Spahn vom 13.03.2020 [4]. In der Folge waren vielfältige personelle und organisatorische Umstrukturierungen notwendig, die an verschiedenen Orten in unterschiedlicher Ausprägung und Geschwindigkeit verliefen. Auch die zu diesem Zeitpunkt sehr unterschiedliche Inzidenz der SARS-CoV-2-Infektion in den einzelnen Bundesländern sowie die damalig ausbleibende Überlastung des Gesundheitssystems mit COVID-19-Erkrankten könnte diese Effekte erklären. In Einrichtungen mit einem geringen Anfall von COVID-19-Erkrankten könnte die Arbeitsbelastung der ärztlichen Mitarbeiter durch den Wegfall der elektiven Patientenversorgung möglicherweise damit sogar gesunken sein. In der Folge könnten hierdurch im Betrachtungszeitraum Zeitressourcen für Forschung und Lehre freigesetzt worden sein. Dies könnte erklären, warum an einigen universitären Klinikstandorten eher eine Verschlechterung, an anderen Orten aber kaum negative Effekte oder sogar eine Verbesserung der Forschungstätigkeiten berichtet wurde.

Dieser Aspekt könnte auch als ein begünstigender Faktor für eine der Pandemie-Situation angepasste Änderung der Forschungsthemen in mehr als der Hälfte der universitären Einrichtungen gewirkt haben. Die Veränderungen der Forschungsthemen wiesen in fast allen Fällen einen thematischen Bezug zur SARS-CoV-2-Pandemie auf. Bereits in der Frühphase der Pandemie planten damit 2/3 der Klinikdirektoren, neue Forschungsanträge mit Bezug zur SARS-CoV-2-Thematik zu stellen. Die universitäre HNO-Heilkunde hat nach den hier erhobenen Daten schon sehr früh versucht, einen Beitrag zur Erforschung der Pandemie zu leisten und damit die Bekämpfung der Infektionswelle zu unterstützen.

Diese Schlussfolgerung lässt sich auch in einer rückblickenden Betrachtung (Stand Dezember 2020) anhand einer Vielzahl bereits publizierter wissenschaftlicher Arbeiten bestätigen. So finden sich zwischenzeitlich eine Reihe SARS-CoV-2-bezogener Publikationen aus oder unter wesentlicher Mitwirkung der befragten Universitätskliniken. Inhaltlich wurde in diesen Publikationen ein breites Spektrum an Themen bearbeitet. Hierzu zählten organisatorische Aspekte, wie Veränderungen der Prozessabläufe [5–7], aber auch fachspezifische Themen, wie die Behandlung onkologischer Patienten [8] oder Cochlea-Implantat-Patienten [9] unter Pandemiebedingungen. Die Durchführung einer Tracheotomie bei an COVID-19 erkrankten Patienten stellte ebenfalls einen wichtigen Inhalt gleich mehrerer Arbeiten dar [10–12]. Klinische Berichte zur Riechstörung betroffener Patienten beleuchteten zudem die organspezifischen Auswirkungen der Erkrankung [13, 14]. Aber auch epidemiologische Themen, wie das Auftreten von COVID-Fällen unter HNO-Ärzten [15], infektiologische Grundlagenarbeiten, wie z. B. die Entstehung und Verteilung von Aerosolpartikeln [16, 17], oder auch Interventionsstudien zum Effekt von Mundspülungen auf die Viruslast [18] wurden bearbeitet. Ebenfalls wurden Publikationen zur Einführung digitaler Medien für die Patientenversorgung [19] und die studentische Lehre [20, 21] erstellt. Diese thematische Vielfalt demonstriert eindrücklich sowohl die hohe Flexibilität als auch den enormen Wert des translationalen klinisch-universitären Wissenschaftssystems, das es ermöglicht, aktuelle medizinische Themen aufzugreifen, wissenschaftlich aufzuarbeiten und sowohl der wissenschaftlichen Community als auch der Patientenversorgung zur Verfügung zu stellen.

Im Hinblick auf die Auswirkungen der Pandemie auf die Forschungstätigkeit einer Universitätsklinik sind prinzipiell unterschiedliche Effekte zu betrachten. Hierzu zählen räumliche, zeitliche, technische, logistische, personelle, finanzielle, regulatorische, inhaltliche oder weitere Aspekte. Die erhobenen Daten zeigen nur in wenigen Fällen, dass es zu einer Reduktion der räumlichen Ressourcen und damit einer Verminderung von Forschungsflächen kam. Die verbleibenden Ursachen wurden in der Umfrage nicht als Einzelvariablen, sondern als „Summationseffekt“ erfasst.

Insgesamt wurden die Folgen der Pandemie überwiegend negativ beurteilt, sodass fast 2/3 aller universitären HNO-Kliniken die Durchführung bereits laufender Forschungsprojekte gefährdet sahen. Die Auswirkungen wurden für klinische Forschungsprojekte schwerwiegender beurteilt als für laborexperimentelle Forschungsprojekte. Eine mögliche Erklärung hierfür wäre die Beschränkung und Priorisierung im Bereich der Patientenversorgung mit starker Prüfung der Notwendigkeit klinischer Untersuchungen und Behandlung sowie die Reduktion von Folgeuntersuchungen durch Kontaktbeschränkungen. Laborexperimentelle Forschungstätigkeit würde aufgrund des in der Regel patientenunabhängigen Ansatzes hiervon weniger stark beeinträchtigt sein. Die Betreuung von Doktoranden war bei den meisten Befragten erschwert, jedoch in keinem Fall unmöglich. Allerdings war die Spannbreite der Ergebnisse sehr groß, d. h. auch wenn mehrheitlich deutliche Beschränkungen angegeben wurden, führten die veränderten Rahmenbedingungen in Einzelfällen auch zu Verbesserungen.

Die Beantragung neuer Forschungsprojekte wurde insgesamt im Beobachtungszeitrum als nicht beeinträchtigt eingeschätzt (Median: 0,0). Die Gründe für diese Effekte wurden im Rahmen der Umfrage nicht spezifisch untersucht. In Zusammenschau mit den bereits vorliegenden Ergebnissen aus dem klinischen Bereich [3] und dieser Umfrage lassen sich Verbesserungen der Forschungssituation möglicherweise auf vergrößerte zeitliche Ressourcen durch die Reduktion der klinischen Routinetätigkeiten, Verschiebung der Aufgabenzuordnung und Änderungen in den Arbeitsformen begründen. Nachweislich waren während des Erhebungszeitraums die klinischen und insbesondere operativen Kapazitäten der Kliniken reduziert [3]. Eine hieraus resultierende Freistellung von Mitarbeitern oder die Einführung von Homeoffice-Zeiten könnten an einigen Einrichtungen auch zu einer Verbesserung der Forschungsbedingungen gegenüber der Situation vor der SARS-CoV-2-Pandemie geführt haben. Diese Interpretation wird durch das an annähernd der Hälfte der Kliniken gesteigerte Interesse und Engagement ihrer Weiterbildungsassistenten an wissenschaftlicher Arbeit gestützt. Individuell beeinflusst wurden diese Effekte wahrscheinlich auch durch Unterschiede in den regionalen Inzidenzraten der SARS-CoV-2-Fälle, aber auch durch die Größe, die Personalstruktur und ggf. regional unterschiedliche Versorgungsaufträge der einzelnen Universitätskliniken. Es erscheint insgesamt plausibel, dass die Auswirkungen der SARS-CoV-2-Pandemie im Hinblick auf die Forschungstätigkeit – im Gegensatz zur klinischen Tätigkeit [3] – nicht nur zu Verschlechterungen, sondern auch zu einer Verbesserung der Arbeitsbedingungen geführt haben. Dies ist ein erfreuliches Ergebnis, da es ein großes wissenschaftliches Potenzial an den universitären HNO-Kliniken aufzeigt. Allerdings wird hier auch deutlich, dass die drei universitären Aufgabengebiete Krankenversorgung, Forschung und Lehre um limitierte Ressourcen konkurrieren. Eine logische Konsequenz aus den im Rahmen dieser Studie erhobenen Daten zur Verstetigung positiver Effekte auf die Forschung wäre daher eine dauerhaft verbesserte Zuweisung von ärztlich-wissenschaftlichen Personalressourcen.

Neben der Forschung besteht eine weitere wesentliche Aufgabe der universitären HNO-Kliniken in der Durchführung der studentischen Lehre. In der Phase des ersten Lockdowns im März und April 2020 zeigten die hier erhobenen Daten an allen 39 HNO-Universitätskliniken eine Einschränkung der Präsenzlehre. Diese Einschränkung beruhte mit hoher Wahrscheinlichkeit auf den initial ausgesprochenen restriktiven Hygienebestimmungen und Kontaktbeschränkungen in den Einrichtungen. Gerade in klinischen Fächern, wie der HNO-Heilkunde, waren daher patientenbasierte Lehrveranstaltungen (z. B. „Unterricht am Krankenbett“ oder „Untersuchungskurse“) in besonderem Maße betroffen. In fast der Hälfte der Universitätskliniken wurden auch Prüfungen nicht mehr als Präsenzveranstaltung durchgeführt.

Als ausgesprochen positiv kann vermerkt werden, dass an nahezu allen Einrichtungen infolge der SARS-CoV-2-Pandemie kurzfristig neue Lehrformate eingeführt wurden und hierzu Präsenzvorlesungen, Präsenzseminare und Unterricht am Krankenbett durch Online-Vorlesungen, -Seminare und -Unterricht ersetzt wurden. Dieses Ergebnis belegt die hohe Flexibilität der universitären HNO-Kliniken, in einer Krisensituation innovative Lösungen zur Sicherung der studentischen Lehre und damit der Sicherung der Ausbildung des ärztlichen Nachwuchses zu entwickeln. Dies ist umso beachtenswerter angesichts des Umstandes, dass fast ein Drittel der befragten Klinikdirektoren mangelnde Ressourcen zur Einführung neuer Lehrformate angeben, die vornehmlich zeitliche, technische und personelle Ressourcen sowie die technische Qualifikation der Mitarbeiter in der Handhabung neuer digitaler Lehrangebote betrafen. Diese Ergebnisse decken sich in Teilen mit der Untersuchung von Offergeld und Mitarbeitern [20], die ebenfalls vorbestehende, z. B. strukturelle Restriktionen beschreiben, welche einer optimalen Umsetzung einer digitalen Lehre entgegenstehen. Dies weist sicher auf einen „Nachholbedarf“ im Hinblick auf notwendige Ressourcen zur Umstellung auf die digitale Lehre hin. Dennoch kann festgestellt werden, dass die in unserer Studie erhobenen Ergebnisse ein positives Bild von der kurzfristig notwendigen, erfolgreichen Umsetzung innovativer Lehrkonzepte durch die universitären HNO-Kliniken zeichnen.

Auch die Weiterbildung ärztlicher Mitarbeiter stellt eine besondere Aufgabe der universitären HNO-Kliniken dar. Die erfolgreiche Durchführung einer Weiterbildung wird auch durch den Erwerb von Kenntnissen in der Diagnostik und Therapie von fachspezifischen Erkrankungen bestimmt, die im Wesentlichen praktische Patientenbehandlungen voraussetzt. Die Auswirkungen der SARS-CoV-2-Pandemie während der Zeit von März und April 2020 könnten damit auch potenziell die Weiterbildung betreffen. Zum Zeitpunkt der Befragung sah die weit überwiegende Mehrheit der Klinikdirektoren die erfolgreiche Weiterbildung der Assistenzärzte allerdings nicht gefährdet. Die Nutzung digitaler Ausbildungsanteile könnte auch im Rahmen der Facharztweiterbildung einen innovativen Ansatz darstellen. Befragt zu einer möglichen Einführung neuer Lehr- und Unterrichtsformate im Rahmen der fachärztlichen Weiterbildung wurde diese von der weit überwiegenden Mehrheit der Klinikdirektoren als grundsätzlich geeignet angesehen. Besonders online verfügbare Lehrmaterialien, Lehrplattformen, Unterricht per Videokonferenz und Online-Seminare wurden positiv bewertet und könnten damit über die Pandemie hinaus zukünftig die Weiterbildung ergänzen bzw. in Krisensituationen sicherstellen. Analog zur Integration digitaler Unterrichtsmethoden in die studentische Lehre könnte diese auch fest in das Weiterbildungscurriculum integriert werden. Diese Aspekte betreffen allerdings vor allem theoretische Lehrinhalte und keine praktisch-diagnostischen oder praktisch-chirurgischen Weiterbildungsinhalte.

## Limitationen der Studie

Die Beurteilung der Ergebnisse unserer Studie muss vor dem Hintergrund der zeitlichen Befristung des Beobachtungszeitraums auf einen Monat im Frühjahr 2020 während der ersten Pandemiewelle betrachtet werden. Besonders die im Verlauf des Sommers 2020 zunächst einsetzende Normalisierung der Tätigkeit der universitären Kliniken und dann die stärkere zweite Infektionswelle mit Beginn im Herbst 2020 hatten zweifelsohne im weiteren Verlauf einen Einfluss auf die Effekte der SARS-CoV-2-Pandemie auf die Forschung, Lehre und die Weiterbildung der befragten Einrichtungen. Es ist daher wahrscheinlich, dass eine Befragung der Klinikdirektoren zu einem späteren Zeitpunkt des Jahres zumindest in Teilen andere Ergebnisse erbracht hätte, als die hier präsentierte, im Mai 2020 abgeschlossene Erhebung.

Im Dezember 2020 wurde der zweite „harte Lockdown“ zum 16.12.2020 umgesetzt, sodass hieraus weitere Einflüsse auf die Tätigkeit der Universitätskliniken zu erwarten sind. Da sowohl die Dauer als auch der Verlauf der Pandemie derzeit kaum vorhersagbar sind, können die im Mai 2020 erhobenen Daten nur als eine Momentaufnahme betrachtet werden.

Die Teilnahme an der Umfrage war auf die Person des Klinikdirektors begrenzt. Die Beurteilung der Auswirkungen der Pandemie auf die im Rahmen dieser Erhebung erfassten Aspekte war subjektiv und auf eine Person an dem jeweiligen Standort beschränkt. Es könnten daher durchaus Abweichungen zwischen der Beurteilung der Situation durch den Klinikdirektor im Vergleich zu anderen Mitarbeitern der jeweiligen Klinik existieren. Auch wenn damit auf methodische Limitationen unserer Studie hingewiesen werden muss, können die hier präsentierten Daten dennoch als eine wichtige erste Standortbestimmung zu den Auswirkungen der SARS-CoV-2-Pandemie auf die universitäre HNO-Heilkunde im Bereich der Forschung, Lehre und Weiterbildung betrachtet werden.

## Fazit

Die Einschränkungen im Bereich der Forschung und Lehre an den universitären Kliniken für Hals-Nasen-Ohren-Heilkunde in der Anfangszeit der Pandemie in Deutschland waren tiefgreifend und zeigten sehr unterschiedliche Auswirkungen. Etablierte bisherige Arbeitsabläufe wie die Präsenzlehre konnten nicht mehr uneingeschränkt durchgeführt werden. Dennoch ließen sich auch positive Auswirkungen der SARS-CoV-2-Pandemie erheben. Die im Frühjahr 2020 plötzlich notwendigen Umstrukturierungen wurden genutzt, um neue, vornehmlich SARS-CoV-2-bezogene Forschungsthemen aufzugreifen und innovative Lehrformate umzusetzen. Somit könnte die Krise auch in einer rückwärtigen Betrachtung als ein „Trigger“ zu Innovationen im Bereich der Forschung und Lehre wahrgenommen werden. Eine abschließende Beurteilung der Auswirkungen ist gegenwärtig (Stand Dezember 2020) – insbesondere vor dem Hintergrund des neuerlichen „Lockdowns“ mit über mehrere Wochen konstant hohen Infektionszahlen, knappen Intensivkapazitäten und erneuter Einschränkung der Elektivbehandlungen von Patienten – nicht möglich. Dies wird zukünftigen Untersuchungen vorbehalten bleiben. Die präsentierten Ergebnisse der Untersuchung zu den Auswirkungen der Pandemie aus dem Frühjahr 2020 haben aber eindrücklich das große innovative Potenzial, die schnelle Reaktionsfähigkeit und die Kreativität der universitären HNO-Kliniken zur Aufrechterhaltung ihrer Forschungs- und Lehrtätigkeit und damit der Sicherung der Ausbildung des ärztlichen Nachwuchses und der fachärztlichen Weiterbildung belegt.

## References

[1] Zhu N, Zhang D, Wang W, Li X, Yang B, Song J (2020). A novel SARS-CoV-2virus from patients with pneumonia in China, 2019. N Engl J Med.

[2] https://coronavirus.jhu.edu/map.html. Zugegriffen: 13.01.2021

[3] Stöver T, Dazert S, Hoffmann TK, Plontke SK, Ambrosch P, Arens C, Betz C, Beutner D, Bohr C, Bruchhage KL, Canis M, Dietz A, Guntinas-Lichius O, Hagen R, Hosemann W, Iro H, Klussmann JP, Knopf A, Kramer S, Lang S, Leinung M, Lenarz T, Löwenheim H, Matthias C, Mlynski R, Olze H, Park J, Plinkert P, Radeloff A, Rotter N, Rudack C, Bozzato A, Schipper J, Schrader M, Strieth S, Stuck BA, Volkenstein S, Westhofen M, Wolf G, Wollenberg B, Zahnert T, Zenk J (2020). Auswirkungen der SARS-CoV-2-Pandemie auf die universitäre Hals-Nasen-Ohrenheilkunde im Bereich der Krankenversorgung. Laryngorhinootologie.

[4] https://www.aerzteblatt.de/nachrichten/111050/Corona-Spahn-verspricht-Krankenhaeusern-finanzielle-Hilfe. Zugegriffen: 13.01.2021

[5] Weiss R, Loth A, Guderian D, Diensthuber M, Kempf V, Hack D, Wicker S, Ciesek S, Graf J, Stöver T, Leinung M (2020). Implementierung eines Betriebskonzeptes in einer HNO-Klinik im Rahmen der SARS-CoV-2-Pandemie. Laryngorhinootologie.

[6] Lüers JC, Klußmann JP, Guntinas-Lichius O (2020). Die COVID-19-Pandemie und das HNO-Fachgebiet: Worauf kommt es aktuell an?. Laryngorhinootologie.

[7] Stuck BA, Dennler U, Hoffmann TK (2020). Präoperative Coronavirustestung in Deutschland : Erfahrungsberichte zum Umgang mit den Empfehlungen der Deutschen Gesellschaft für Hals-Nasen-Ohrenheilkunde, Kopf- und Hals-Chirurgie e. V. HNO.

[8] Hoffmann TK, Greve J, Laban S, Schuler PJ (2020). Besonderheiten in der Behandlung von Patienten mit Kopf-Hals-Karzinomen in Zeiten der COVID-19-Pandemie. HNO.

[9] Aschendorff A, Arndt S, Kröger S, Wesarg T, Ketterer MC, Kirchem P, Pixner S, Hassepaß F, Beck R (2020). Qualität der Cochleaimplantat-Rehabilitation unter COVID-19-Bedingungen. HNO.

[10] McGrath BA, Brenner MJ, Warrillow SJ, Pandian V, Arora A, Cameron TS, Añon JM, Hernández Martínez G, Truog RD, Block SD, Lui GCY, McDonald C, Rassekh CH, Atkins J, Qiang L, Vergez S, Dulguerov P, Zenk J, Antonelli M, Pelosi P, Walsh BK, Ward E, Shang Y, Gasparini S, Donati A, Singer M, Openshaw PJM, Tolley N, Markel H, Feller-Kopman DJ (2020). Tracheostomy in the COVID-19 era: global and multidisciplinary guidance. Lancet Respir Med.

[11] Pudszuhn A, Voegeler S, Berger C, Treskatsch S, Angermair S, Hansen S, Hofmann VM (2020). Elektive Tracheostomie bei COVID-19-Patienten – Erfahrungen mit einem standardisierten interdisziplinären Vorgehen. HNO.

[12] Kempfle JS, Löwenheim H, Huebner MJ, Iro H, Mueller SK (2020). Management von Patienten mit Tracheostoma während der COVID-19-Pandemie: Literaturüberblick und Demonstration. HNO.

[13] Otte MS, Klußmann JP, Luers JC (2020). Riechstörungen bei COVID-19 – aktueller Wissensstand. Laryngorhinootologie.

[14] Bocksberger S, Wagner W, Hummel T, Guggemos W, Seilmaier M, Hoelscher M, Wendtner CM (2020). Temporäre Hyposmie bei COVID-19-Patienten [Temporary hyposmia in COVID-19 patients. HNO.

[15] Herzog M, Beule AG, Lüers JC, Guntinas-Lichius O, Sowerby LJ, Grafmans D (2020). Results of a national web-based survey on the SARS-CoV-2 infectious state of otorhinolaryngologists in Germany. Eur Arch Otorhinolaryngol.

[16] Loth AG, Guderian DB, Haake B, Zacharowski K, Stöver T, Leinung M (2020). Aerosol exposure during surgical tracheotomy in SARS-coV-2 positive patients. Shock.

[17] Guderian DB, Loth AG, Weiß R, Diensthuber M, Stöver T, Leinung M (2020). In vitro comparison of surgical techniques in times of the SARS-CoV-2 pandemic: electrocautery generates more droplets and aerosol than laser surgery or drilling. Eur Arch Otorhinolaryngol.

[18] Gottsauner MJ, Michaelides I, Schmidt B, Scholz KJ, Buchalla W, Widbiller M, Hitzenbichler F, Ettl T, Reichert TE, Bohr C, Vielsmeier V, Cieplik F (2020). A prospective clinical pilot study on the effects of a hydrogen peroxide mouthrinse on the intraoral viral load of SARS-CoV-2. Clin Oral Investig.

[19] Hagge D, Knopf A, Hofauer B (2020). Chancen und Einsatzmöglichkeiten von Telemedizin in der Hals , Nasen- und Ohrenheilkunde bei der Bekämpfung von SARS-COV-2 : Narratives Review. HNO.

[20] Offergeld C, Ketterer M, Neudert M, Hassepaß F, Weerda N, Richter B, Traser L, Becker C, Deeg N, Knopf A, Wesarg T, Rauch AK, Jakob T, Ferver F, Lang F, Vielsmeier V, Hackenberg S, Diensthuber M, Praetorius M, Hofauer B, Mansour N, Kuhn S, Hildenbrand T (2020). „Ab morgen bitte online“: Vergleich digitaler Rahmenbedingungen der curricularen Lehre an nationalen Universitäts-HNO-Kliniken in Zeiten von COVID-19 : Digitale Lehre an nationalen Universitäts-HNO-Kliniken. HNO.

[21] Kaulitz S, Engert J, Roos C, Filsinger M, König S, Hackenberg S (2020). Digital practical course of otorhinolaryngology and examination technique “to go”. GMS J Med Educ.

